# The Nadir Crater offshore West Africa: A candidate Cretaceous-Paleogene impact structure

**DOI:** 10.1126/sciadv.abn3096

**Published:** 2022-08-17

**Authors:** Uisdean Nicholson, Veronica J. Bray, Sean P. S. Gulick, Benedict Aduomahor

**Affiliations:** ^1^ School of Energy, Geoscience, Infrastructure and Society, Heriot-Watt University, Edinburgh, UK.; ^2^ Lunar and Planetary Laboratory, University of Arizona, Tucson, AZ, USA.; ^3^ Institute for Geophysics and Department of Geological Sciences, University of Texas at Austin, Austin, TX, USA.; ^4^ Center for Planetary Systems Habitability, University of Texas at Austin, Austin, TX, USA.

## Abstract

Evidence of marine target impacts, binary impact craters, or impact clusters are rare on Earth. Seismic reflection data from the Guinea Plateau, West Africa, reveal a ≥8.5-km-wide structure buried below ~300 to 400 m of Paleogene sediment with characteristics consistent with a complex impact crater. These include an elevated rim above a terraced crater floor, a pronounced central uplift, and extensive subsurface deformation. Numerical simulations of crater formation indicate a marine target (~800-m water depth) impact of a ≥400-m asteroid, resulting in a train of large tsunami waves and the potential release of substantial quantities of greenhouse gases from shallow buried black shale deposits. Our stratigraphic framework suggests that the crater formed at or near the Cretaceous-Paleogene boundary (~66 million years ago), approximately the same age as the Chicxulub impact crater. We hypothesize that this formed as part of a closely timed impact cluster or by breakup of a common parent asteroid.

## Teaser

Seismic data reveal a candidate impact crater offshore West Africa that formed close in time to the K-Pg extinction event.

## INTRODUCTION

Hypervelocity impacts of large asteroids or comets with Earth are still poorly understood despite the risk they pose. Hazardous impactors of ~50 m in diameter, equivalent to the Tunguska event in 1908 (*1*), collide with Earth every ~900 years, and those larger than 1 km, with potential catastrophic regional or global consequences, every ~1 million years (*2*). The largest confirmed impact events have had globally catastrophic consequences, with the 66–million-year-old (Ma) Chicxulub event causing one of the most important extinction events in Earth history, at the Cretaceous-Paleogene (K-Pg) boundary (*3*, *4*). Despite their frequency, only a small proportion of hypervelocity impacts have been preserved or discovered, with approximately 200 confirmed craters on Earth (*5*). Marine target impacts should constitute the majority of craters on Earth, given that most of Earth’s surface (~71% currently) is covered in water. However, only 15 to 20 confirmed hypervelocity impact craters were marine impact events (*6*). Most of these were in shallow epicontinental seas, the majority of which are now exposed onshore and only partially preserved. The general lack of well-preserved marine craters at different target water depths means that both the physical characteristics of these craters and the consequence of these events outside of the crater itself (ejecta distribution, seismicity, and tsunami generation) is typically unknown and based primarily on numerical models (*7*, *8*). Lack of well-preserved and well-imaged craters and associated deposits limits our ability to validate these numerical models, particularly for intermediate-to-deep water impact sites.

Marine target impacts are not the only events that may be underrepresented in the geological record. Despite the common occurrence of binary (gravitationally coupled) asteroids in near Earth orbit (~15%), only a small number (2 to 4%) of confirmed craters are recognized as potential binary impact structures (*9*). The number of binary impact structures may, in fact, be even smaller than proposed as a number of these candidates have subsequently been disputed or disproven (*10*). By contrast, on Venus, statistical analysis of the distribution of “dark splotches” formed by atmospheric disintegration of asteroids indicates that the number of binary impacts is approximately 14% of total impact events (*11*), although we note that the atmospheric perturbations documented by these dark splotches may not result in two distinct craters at the surface. However, it does suggest that binary asteroid flux into the atmosphere at least may be underestimated and that binary impacts may be underrepresented in the cratering record on Earth.

As well as binary impacts, there are also cases of possible impact clustering, where several craters formed within a short time period, typically up to several million years in duration. The only confirmed case of impact clustering on Earth is in the Ordovician, hypothesized to be related to an L-chrondrite parent asteroid breakup event (*12*). This cluster is documented both by an increased terrestrial cratering rate, constrained by Ar-Ar dating of impactites (*12*), and evidence for an elevated flux of extraterrestrial dust across the same >2-Ma period (*13*). A number of other possible clusters in the Cretaceous and Eocene have also been proposed (*5*), but a lack of sufficiently robust, high-precision dates for individual craters means that these are not yet confirmed. These clusters, if present, are likely formed by multiple impacts from genetically related “asteroid families,” which form as a result of collision and breakup of larger parent bodies within the asteroid belt—a process that continues to the present day (*14*). Although the evidence for these events is rare on Earth, impact clustering is observed on other planetary bodies including the Moon (*15*). These events have been linked to periods of major environmental perturbation on Earth (*13*, *15*), suggesting that impact clusters on Earth may be an important hazard despite the lack of a preserved terrestrial record.

**Fig. 1.**
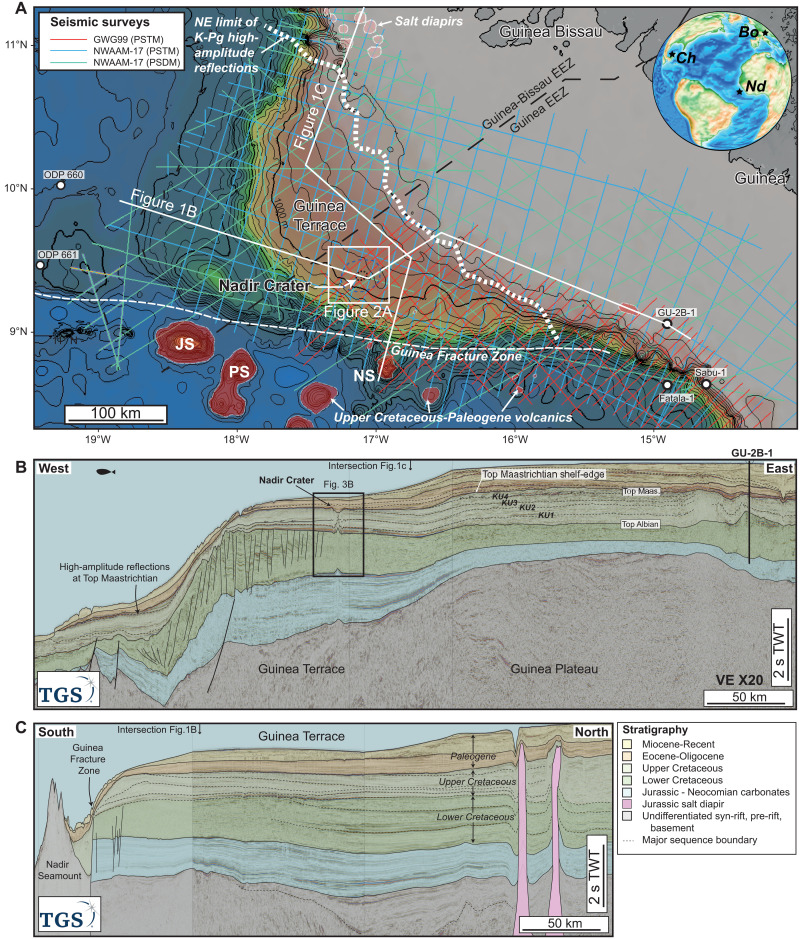
Map and regional seismic sections showing location of Nadir Crater. (**A**) Regional bathymetry map of the Guinea Plateau and Guinea Terrace showing location of 2D seismic reflection and well data used in this study. JS, Jane Seamount; NS, Nadir Seamount; PS, Porter Seamount. The white dashed line shows the NE extent of high-amplitude discontinuous seismic facies at the top Maastrichtian interpreted as ejecta deposits and associated tsunami deposits. The north-east limit of this facies closely corresponds with the Maastrichtian shelf-slope break at the landward margin of the Guinea Terrace. Inset map shows a paleogeographic reconstruction of the Atlantic near the end of the Cretaceous, ~66 Ma ago, made using GPlates software (*58*). Ch, Chicxulub Crater; Nd, Nadir Crater; Bo, Boltysh Crater. (**B**). Regional composite 2D seismic reflection profile extending from the GU-2B-1 well in the east to the deep Atlantic basin in the west, showing the structural and stratigraphic character of the Guinea Plateau and Guinea Terrace. (**C**) North-South seismic profile from the salt basin in the north to the Nadir Seamount, south of the Guinea Fracture Zone. Data courtesy of the Republic of Guinea and TGS.

In this paper, we use an extensive two-dimensional (2D) seismic reflection dataset from the Guinea Plateau, West Africa, to present evidence for a previously unidentified candidate complex impact crater that we refer to as the Nadir Crater, named after the Nadir Seamount, situated 100 km to the south (Fig. 1). Seismic data allow us to construct a robust seismic stratigraphic framework for the Guinea Plateau and, hence, the approximate age and paleoenvironment of the crater. We use numerical models to simulate the impact event, test whether the crater geometry is consistent with an impact origin, and constrain the probable water depth. We combine seismic observations and model outcomes to build a conceptual model of crater formation and the potential environmental consequences of such an event. We then consider the association of this possible impact with other terrestrial events of similar age.

### Geological setting: The Guinea Plateau

The Guinea Plateau is an area of thinned continental crust that extends ~400 km west of the coast of Guinea and Guinea Bissau to the continental slope of West Africa (Fig. 1). The plateau currently consists of a shallow (<200-m water depth) eastern segment and an intermediate (~200 to 1200 m deep) western segment known as the Guinea Terrace. The plateau is separated from the deep (2.5 to 4 km) ocean basin to the south by the Guinea Fracture Zone. There are several large seamounts on the ocean crust to the south of the Guinea Fracture Zone, the closest of which is the Nadir Seamount.

The Guinea Plateau formed following the Triassic-Jurassic rifting, with the final breakup between the conjugate margins of West Africa and North America in the Middle-Late Jurassic (*16*). The plateau was then part of the Central Atlantic passive margin of West Africa during the Late Jurassic to Early Cretaceous. Restricted marine conditions occurred in the early post-rift stage, including the deposition of extensive salt deposits in the basins to the north of the Guinea Plateau, and a carbonate platform subsequently developed across the region up until the Barremian (~125 to 129 Ma ago) (*17*). Marine and continental clastic deposition continued in a passive margin setting until the end of the Albian (~100 Ma ago). During the late Albian, the southern end of the plateau was affected by transform tectonics during the final breakup of Southern Gondwana—the separation of South America and Africa. This resulted in the formation of large strike-slip faults and subaerial erosion at the southern plateau margin, leading to the formation of a prominent regional unconformity (*17*). The Guinea Plateau, and particularly the extended continental crust of the Guinea Terrace, rapidly subsided following the breakup event and has continued to subside to the present day as the lithosphere has aged and cooled.

## RESULTS AND DISCUSSION

### K-Pg stratigraphy of the Guinea Terrace

The proposed Nadir Crater is located on the southwest Guinea Terrace, ~60 km north of the Guinea Fracture Zone and ~100 km NNW of the Nadir Seamount (Fig. 1). The current water depth in this location is around 900 m, and the crater is covered by 300 to 400 m of marine sediment.

**Fig. 2.**
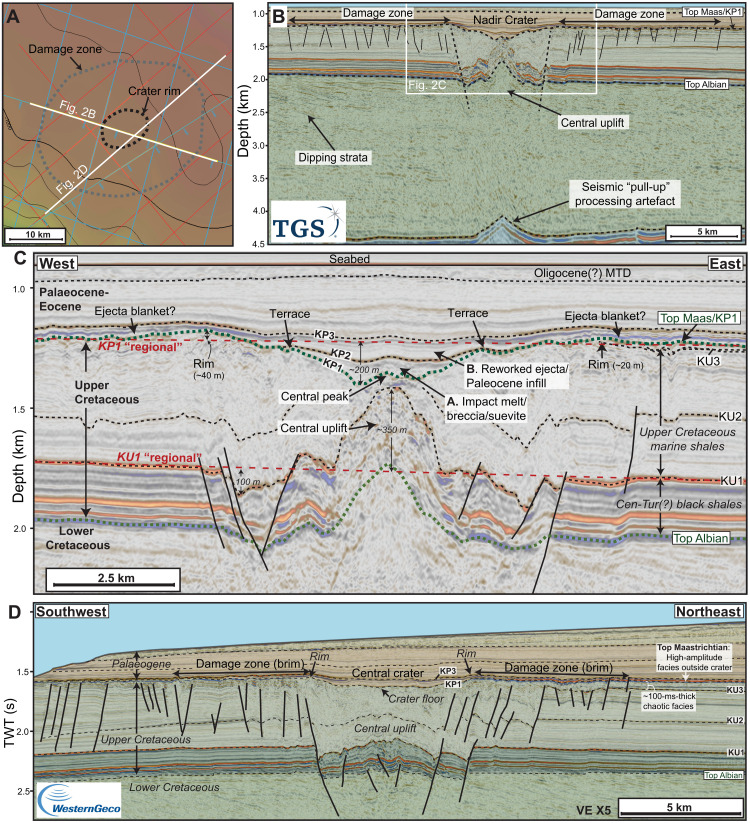
Seismic characteristics of the Nadir Crater. (**A**) Seabed depth map of crater showing seismic line locations and the mapped extent of the crater rim and damage zone. (**B**) W-E seismic section (pre-stack depth migration – depth domain) across the crater, highlighting the crater morphology and damage zone, and the extent of subsurface deformation. Data courtesy of the Republic of Guinea, TGS and WesternGeco. Stratigraphic key is on Fig. 1. (**C**) Detailed seismic stratigraphic and structural elements of the crater. KP, Cretaceous-Paleogene sequence (KP1 equivalent to Top Maastrichtian); KU, Upper Cretaceous seismic horizons. KU1 and KP1 “regionals” are schematic reconstructions of these seismic horizons before formation of the crater at the end of the Cretaceous and are used to reconstruct a conceptual model of crater formation (Fig. 5). (**D**) SW-NE seismic section (pre-stack time migration – time domain) across the crater, showing crater morphology and seismic facies outside the crater, including high-amplitude seismic facies sitting above a ~100-ms-thick unit of chaotic reflections, interpreted to have formed as a result of seismic shaking following the impact event. Data courtesy of the Republic of Guinea and WesternGeco Multiclient.

The sequence stratigraphic framework for the Guinea Terrace is constrained by biostratigraphic ages from the GU-2B-1 and Sabu-1 wells and is consistent with previous interpretations of the south of the Guinea Plateau (*17*). The Cretaceous stratigraphy of the Guinea Terrace is separated into a pre- and syn-transform Lower Cretaceous (~145 to 100 Ma ago) sequence and a posttransform Upper Cretaceous (~100 to 66 Ma ago) sequence, separated by the regional Top Albian unconformity (Fig. 1, B and C). The Upper Cretaceous sequence consists of a series of largely undeformed, laterally continuous reflections across the Guinea Terrace. These include a set of prominent seismic reflections (KU1–KU4) that are interpreted as major flooding surfaces (Fig. 1B). In terms of seismic facies, the Upper Cretaceous can be divided into two distinct units (Fig. 2). The lower unit (Top Albian to KU1) consists of a 300-m-thick series of very-high-amplitude reflections (Fig. 2) that can be correlated laterally across the Guinea Terrace. The upper unit (KU1 to Top Maastrichtian/KP1) consists of a ~500-m sequence of moderate- to low-amplitude seismic reflections. The main seismic reflections KU1–KU4 are characterized as downlap surfaces that separate individual prograding clinoform sequences (Fig. 1B), which evidently formed during discrete marine transgressive-regressive events during the Late Cretaceous. The height of individual clinoform sets, from the offlap break (shelf edge) to the downlap surface (base of slope), ranges from 250 to 500 m, providing constraints on water depth for discrete time intervals. At the Top Maastrichtian (~66 Ma ago), the shelf-edge is located near the boundary between the shallow Guinea Plateau and Guinea Terrace, with water depths in the Guinea Terrace, including the Nadir Crater site, likely exceeding 500 m.

Although the Top Cretaceous (Top Maastrichtian) reflection is well constrained, the precise age of the intra-upper Cretaceous reflections KU1–KU4 remains uncertain because of the low amplitude, laterally discontinuous seismic character, and poor seismic quality in the eastern plateau near the exploration wells. The KU1 reflector is tentatively dated as being near top Turonian (~90 Ma ago) in age, both by correlation with the GU-2B-1 well and from the equivalent seismic facies on the conjugate margin in the Demerara Plateau (*18*). The distinct high-amplitude seismic facies between the top Albian and KU1 reflector on the Guinea Terrace likely represent black shale deposits with elevated total organic carbon (TOC), equivalent to those found on the conjugate tectonic margin (*19*). The sequence above, between the KU1 and Top Maastrichtian reflectors, likely consists of relatively low TOC marine shale and marl, based on the wells in Guinea and on the Demerara Plateau.

The Top Maastrichtian horizon is interpreted at the top of a set of distinctive, high-amplitude reflections and represents both an erosional unconformity on the SW Guinea Terrace and a correlative conformity farther to the north and east (Fig. 1, B and C). These high-amplitude reflections have a discontinuous reflection character that extends across the entire Guinea Terrace and in deep water but is locally absent (eroded) on the continental slope and shallow Guinea Plateau. The reflection character of the Top Maastrichtian is also different on the shallow Guinea Plateau, where it corresponds to a moderate-amplitude, continuous seismic reflection (Fig. 1B). The high-amplitude Top Maastrichtian reflection on the Guinea Terrace sits above a ~100-ms-thick unit of chaotic seismic facies with discontinuous, disturbed reflections (Fig. 2D). This sequence stratigraphic framework places the Nadir Crater at or close to the Top Maastrichtian, approximately 66 Ma.

The overlying Cenozoic sequence (<66 Ma ago) includes a thick sequence of marine carbonates and clastics in the eastern plateau (*17*), but this sequence thins considerably across the Guinea Terrace (Fig. 1B). Although there is some uncertainty over the precise correlation of the Cenozoic sequence boundaries, the Neogene sequence (<23 Ma ago) is very thin, or absent, on the Guinea Terrace. In this location, there is a ~300-m-thick Paleocene-Eocene sequence (~34 to 66 Ma ago) and a series of Mass Transport Deposits of probable Oligocene age (~23 to 34 Ma ago) immediately below the seabed (Fig. 2), which suggests a continuation of relatively deep-water conditions throughout the Cenozoic.

### Crater morphology and the impact crater hypothesis

The proposed Nadir Crater consists of an ≥8.5-km-wide, concave-up depression at the Top Maastrichtian horizon (K-Pg boundary surface; locally referred to as KP1 for the expanded sequence in the crater). It is evident on two seismic profiles (Fig. 2), suggesting that it is circular or elliptical in planform geometry. We note that the seismic lines may not precisely cross the middle of the apparent crater; therefore, 8.5 km is a minimum diameter, measured from the crater rim (*20*). The crater floor sits 200 m below the “regional” (predeformation seabed; KP1), with the crater rim elevated 20 to 40 m above this surface. There is a subtle central peak in the middle of the structure rising above the relatively flat crater floor (Fig. 2C); this sits above a pronounced uplift in the substrate below, with the Top Albian and KU1 horizons elevated ~350 m above regional. The crater interior also displays a stepped (terraced) morphology near the crater rim. Below the crater rim and terraces, and on either side of the central uplift, there is an area of low seismic reflectivity, with clear evidence of intense deformation—both faulting and extensive folding—extending more than 700 m below the Top Maastrichtian reflection interpreted as the crater floor. Outside of the proposed crater rim, there is also a broader area of faulting, with fault planes dipping toward the crater, extending around 10 to 12 km on either side of the crater (Fig. 2B).

These observations are all consistent with a complex crater with an impact origin. The ratio of crater rim height to crater depth (~1:10), crater depth to width (~1:20), crater diameter to damage zone (~1:2), and the presence of the terraces and magnitude of the central stratigraphic uplift are all consistent with confirmed hypervelocity impact craters elsewhere on Earth (*21*, *22*) and on other terrestrial planets (*23*). A terraced crater interior and central peak is expected for complex craters and is consistent with the predicted morphology for terrestrial impact craters larger than ~2 to 4 km (*24*).

Deformation below the crater floor is also consistent with what would be expected in a complex crater. A central uplift forms during the crater modification stage when shock waves decay to form elastic waves, weakened crater rims collapse, and the shocked substrate below the crater flows vertically upward (*21*). This modification process also results in the formation of a crater rim monocline likely infilled with impactites and consistent with the seismic evidence of intense deformation immediately below the proposed crater floor. The seismic facies in the Nadir Crater indicates both faulting and folding, implying brittle deformation of sediments and sedimentary rocks below the crater floor consistent with acoustic weakening and then an increase in rock strength as the time since impact increases. The location and depth of this low reflectivity zone correspond well to the expected position of a “breccia moat” and the depth of damage expected for an impact crater of this size (*25*). We interpret the 10- to 12-km damage zone outside of the crater rim to have been caused by the response of the surrounding weak sea floor sediments to the impact shockwave and to the collapse of the transient crater during the modification stage (*26*).

The displacement of the central uplift within the upper Cretaceous sequence is broadly consistent with other terrestrial impact craters. Scaling trends for terrestrial impact craters suggest the maximum depth of stratigraphic uplift beneath the center of a 8.5-km-diameter crater would be a depth of 630 to 780 m below the preimpact surface (*22*). In the case of Nadir, uplift is clearly evident down to the Top Albian surface, ~800 m below the preimpact seabed. Below the Albian surface, seismic reflections also show deflection down to the Jurassic (Fig. 1B). However, based on the seismic character, below the Top Albian horizon, this is likely to be a seismic “pull-up” effect due to contrasting seismic velocities. This suggests relatively high velocities below the crater floor, likely a result of impact-related compaction and shock metamorphism in unconsolidated sediments below the seafloor. Increased seismic velocities and density in the central uplift are also observed at the Mjølnir Crater (*27*), where shallow marine clastic sediments formed the target lithologies. By contrast, seismic velocities around the periphery (annular trough) at Mjølnir are lower because of brecciation and associated porosity-increasing processes, similar to that also observed in sedimentary and basement rocks at the Chicxulub impact crater (*28*). At Nadir, impact-related compaction and shock metamorphism in an unconsolidated porous target likely resulted in a decrease in porosity and thus increased relative velocity immediately below the crater floor and in the central uplift. Evidence from other impact sites shows that elevated temperatures and hydrothermal systems can persist for millions of years after impact (*29*), and we suggest that hydrothermal mineralization could also result in increased seismic velocities below the crater floor.

The high-amplitude Top Maastrichtian reflectors around the crater site corresponds to an expanded sequence in the crater floor (Fig. 2C), which we interpret to have formed at or near the K-Pg boundary. This includes several distinct seismic reflections (locally referred to as KP1 to KP3) that separate individual units with distinctive seismic facies within the crater. Above the crater floor, Unit A (between KP1 and KP2) consists of a ~100-m-thick sequence of transparent seismic facies, with a hard (increase in acoustic impedance) top and base. The base reflector truncates the deformed reflections in the underlying sequence. Because of the lack of internal reflections and hard top, we interpret this as a possible suevite unit perhaps equivalent to the unsorted melt-rich suevite observed at Chicxulub [e.g., (*30*–*32*)]. Above this, Unit B (from KP2 to KP1) consists of a low-amplitude sequence of near horizontal reflections that onlap the inner crater walls. This sequence is laterally and temporally equivalent to the high-amplitude facies outside of the crater rim. We interpret this as possible reworked ejecta transported back into the crater in a resurge event following the vaporization and evacuation of water from the crater during the initial impact. These suevite would likely have low seismic velocities and be relatively well sorted because of settling within the reflooded crater as observed in Chicxulub [e.g., (*30*, *32*)].

The distinctive high-amplitude reflections outside of the crater would be consistent with the presence of an ejecta blanket, evacuated from the transient crater on impact. This seismic facies is more extensive than the recorded extent of relatively well-preserved ejecta of terrestrial craters (*33*), and it is noted that impacts into marine environments tend to lack extensive or continuous ejecta deposits when compared to “dry” impacts (*34*). To explain the extent of this high-amplitude layer, we interpret this deposit to be caused by a combination of an ejecta blanket and sediment reworking by the rim wave tsunami induced by the impact (*6*, *8*). This deposit overlies a zone of chaotic seismic facies sitting immediately below the Top Maastrichtian reflector, which may represent partial fluidization and lateral flow of the seabed and shallow substrate caused by the impact shock wave or seismic energy that would arrive well in advance of the tsunami.

The gross architecture of the crater is similar to that observed in confirmed complex marine impact craters. In particular, the presence of a deep “inner” crater with central peak, a flat outer region (brim) above the damage zone (Fig. 2D), is similar to the “inverted sombrero” morphology observed in the Chesapeake (*35*), Lockne (*36*), and Flynn Creek (*37*) craters. In some of these cases, an elevated crater rim cannot be clearly identified, and the outer “brim” is considered to be part of the crater itself. However, for Nadir, the crater rim is largely preserved, and we consider the diameter of the crater to be the area within the rim (*20*).

The morphology, stratigraphy, and structural characteristics all support our impact crater hypothesis for the Nadir Crater. However, the impact crater hypothesis can only be tested by drilling and physical recovery of shocked mineral phases from the crater.

### Alternative crater-forming mechanisms

There are several alternative processes that could generate large-scale (>km) circular crater-like features such as the Nadir Crater. These include subsurface salt withdrawal or dissolution, gas or fluid escape, collapsed volcanic calderas or maars, collapse features associated with carbonate dissolution, polygonal faulting induced by dewatering, the erosive actions of currents and tectonic (strike-slip pull-apart) deformation, or a combination of these processes (*38*–*40*). However, most of these processes either are inconsistent with the local geology and stratigraphy on the Guinea Terrace or would result in crater morphologies and scaling relationships that differ substantially from those observed at Nadir. The most plausible of these alternative mechanisms are discussed below.

The collapse of circular (planform) salt diapirs at the seabed can result in circular depressions with well-developed radial faults (*41*). However, salt withdrawal would not be expected to produce a central uplift or terraces nor the high-amplitude extra-crater facies observed at Nadir. Salt diapirs are evident in the far northwest of the Guinea Terrace (Fig. 2C); however, these are over 250 km away, and there is no evidence of any diapirs cutting across the Mesozoic stratigraphy elsewhere in the wider Guinea Plateau. Neither is there any local evidence for a salt diapir at the site of the crater.

Fluid escape, in some cases in association with mud diapirism/extrusion, is a common feature of many ocean basins, in particular continental margins where methane is abundant. These are circular to elliptical features in the seabed and may superficially have a similar surface expression to the Nadir Crater. Most pockmarks range from a few meters to a few hundred meters in diameter (*38*) but can in some cases can exceed 10 km in diameter (*42*). However, these almost always occur in clusters of several tens to thousands of individual features, and we see no other comparable features anywhere on the Guinea Plateau or any seismic evidence of gas or other fluid escape features. In addition, although pockmarks may be associated with underlying mud volcanoes, or diapirs, they do not exhibit the collapsed annular trough, central uplift, crater rim, and candidate ejecta observed at Nadir; therefore, we do not consider this a likely mechanism of crater formation.

Strike-slip deformation can result in the formation of small rhombohedral to elliptical “pull apart” basins bounded by extensional or transtensional faults. However, these basins are rarely circular in planform and usually have an aspect ratio (length:width) of >2 (*38*). In the case of Nadir, the aspect ratio appears to be close to 1, and other features (rim and central uplift) are not consistent with a strike-slip origin. In addition, although the Nadir Crater is situated relatively close to a transform margin, strike-slip activity along the margin ceased by the Albian or earliest Cenomanian (~100 Ma ago), making a tectonic origin for the basin highly unlikely.

The alternative origin for the Nadir Crater most consistent with local geology is that it may have formed in association with volcanism, in particular a phreatomagmatic eruption, where magma interacts with water in shallow aquifers to form a maar. There is evidence of near-contemporaneous volcanism in the region (Fig. 1). The Nadir Seamount is only slightly younger in age [58.6 Ma; (*43*)] than the Nadir Crater and was formed in association with a long-lived hot spot that later formed the Grimaldi Seamounts, along the Guinea Fracture Zone (*44*). However, maars rarely form crater structures larger than 2 to 3 km in diameter and have a characteristic funnel-like morphology (*45*). The seamounts and other Upper Cretaceous-Paleogene volcanic features on the plateau margin (Fig. 1) all display a convex-up morphology, unlike the Nadir Crater. Therefore, the absence of the characteristic maar morphology and the presence of the central uplift render a volcanic origin for the Nadir Crater unlikely. There are no other features anywhere on the Guinea Plateau or Terrace, in either the salt province in the north or the volcanic province in the south, with a morphology similar to that of the Nadir Crater.

### Hydrocode marine impact simulations

Seismic observations of the Nadir Crater show a compelling set of features consistent with an impact origin in a marine setting. In this section, we present hydrocode model simulations of marine impacts for different water depths. The simulated crater morphology, ejecta distribution, and uplift of subsurface strata are compared to the seismic observations of the Nadir structure to test whether these characteristics are consistent with an impact origin.

**Fig. 3.**
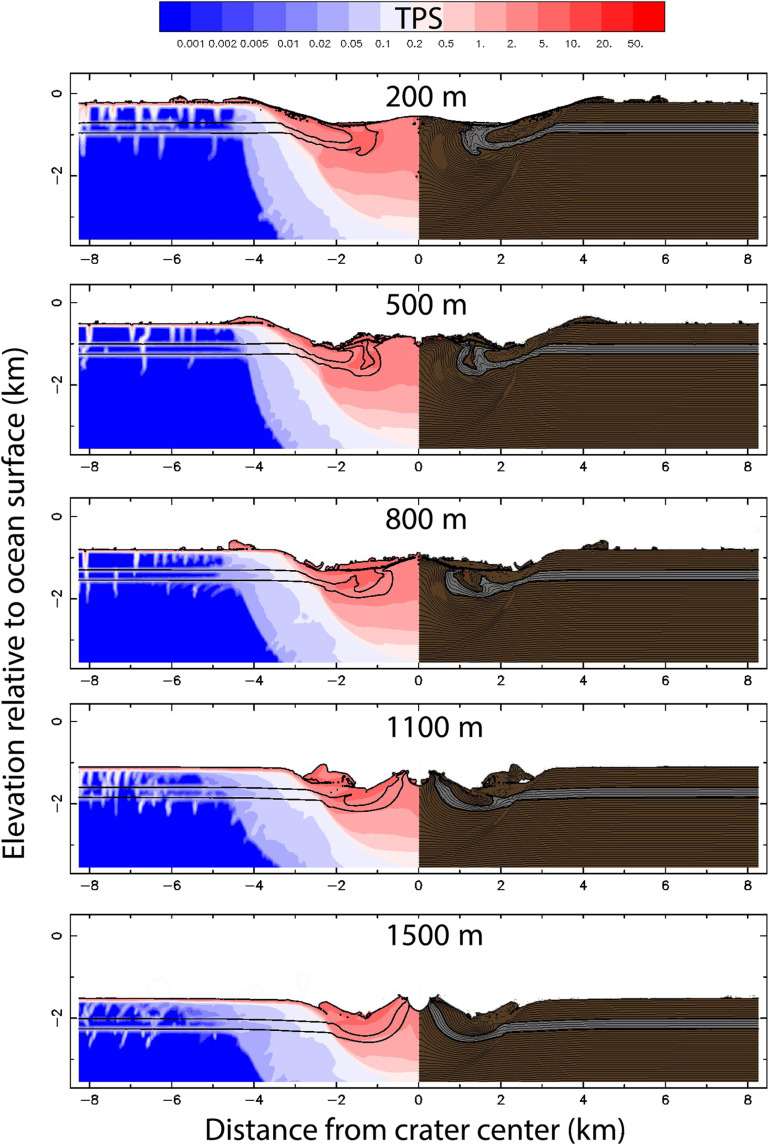
Numerical model results of iSALE hydrocode simulations of final crater architecture for different water depths (200, 500, 800, 1100, and 1500 m, as indicated on the figure) Models assume a 400-m-diameter impactor with an impact angle of 90°. The water layer has been removed from each image to better highlight the final crater morphology. Model outcomes show total plastic strain (TPS) on the left and deformed lithologies on the right—the gray unit represents the assumed Cenomanian-Turonian black shale deposits as a marker horizon. Movies of full simulations are included in the Supplementary Materials.

Hydrocode model results for a 400-m asteroid impact into various water depths above a sedimentary target are shown inFigs. 3 and 4, and full simulations are shown in movies S1 to S5. All models show the formation of a ~7- to 9-km-wide crater (measured rim to rim) with elevated strata in the rim area and a significant central uplift (Fig. 3). The amount of rim collapse that occurs during crater modification increases with ocean depth, with larger water depths facilitating a more marked collapse of the uplifted and overturned beds that comprise the crater rim. The collapsed rim deposit partially fills the original crater cavity, creating a shallow crater and burying the base of the central uplift, resulting in a relatively modest central peak exposed at the surface. Above water depths of 1000 m, all rim and proximal ejecta material is washed back into the crater, depositing as a resurge deposit, further shallowing the crater. In all cases, even when no ejecta remains outside of the crater, the surrounding terrain is tectonically disturbed with multiple faults (Fig. 3).

**Fig. 4.**
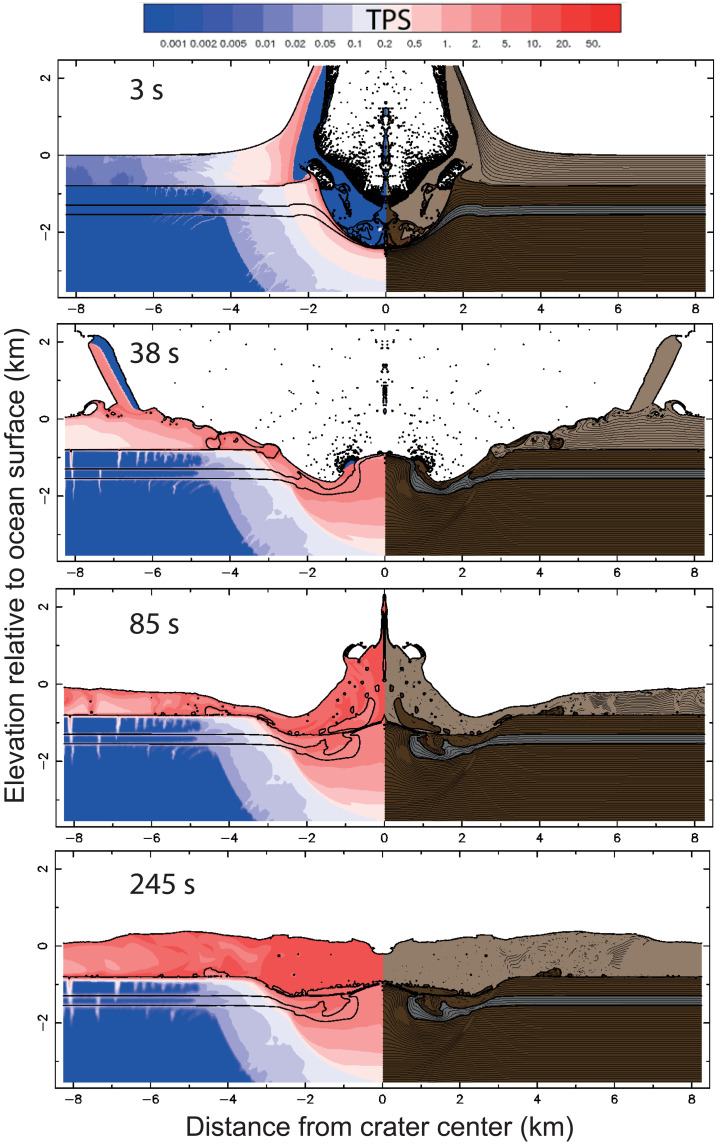
Snapshots of numerical model results of an iSALE hydrocode simulation for 800-m target water depth. We consider this to be the most likely water depth for the impact, based on crater morphology. Other model parameters as for Fig. 3. Model snapshots show transient crater formation and generation of a rim-wave tsunami after 3 s; rebound of central uplift and propagation of a rim-wave tsunami away from crater at 38 s; crater collapse, resurge, and central jet formation at 85 s; and further resurge at 245 s. Movies of full simulations are included in the Supplementary Materials.

A central uplift develops in all models, although the geometry varies significantly with varying water depths between models—the greater the water depth, the larger the central uplift height (relative to preimpact elevation), presumably due to the additional rebound of the exposed rocks after removal of the greater overburden pressure of a deeper ocean. In these models, the stratigraphic uplift that occurs at the crater center raises strata upward from a maximum depth of 2 km beneath the preimpact surface and brings material from >1 km in the subsurface to the top of the central peak (Fig. 3). This is somewhat higher than the total amount of exhumation (~750 m) inferred from seismic observations of the Nadir Crater, although we do not know if the seismic line (Fig. 2) is situated directly over the center of the crater.

Assessing the total plastic strain (TPS)—a nondimensional measure of shear deformation—for all the simulated craters shows a clear region of brecciation and deformation beneath the crater surface. TPS is similar for all water depths, with TPS values >1 to a depth of ~2.5 km below the crater. Elevated TPS values also occur outside of the crater rims, where a series of subvertical faults or fractures form up to at least 5 km on either side of the crater. These extend to around 1 km below the preimpact seabed, consistent with seismic observations for the Nadir Crater (Fig. 2).

These numerical models are consistent with the seismic observations from the Nadir Crater, including the depth of the crater (for deeper water models), development of elevated rims (for models <1000 m) and terraces, the character of the central uplift, and the extent of the damage zone outside of the crater. These numerical simulations thus provide additional support for an impact origin for the Nadir Crater, with water depths of ~800 m most consistent with seismic observations (Fig. 3).

**Fig. 5.**
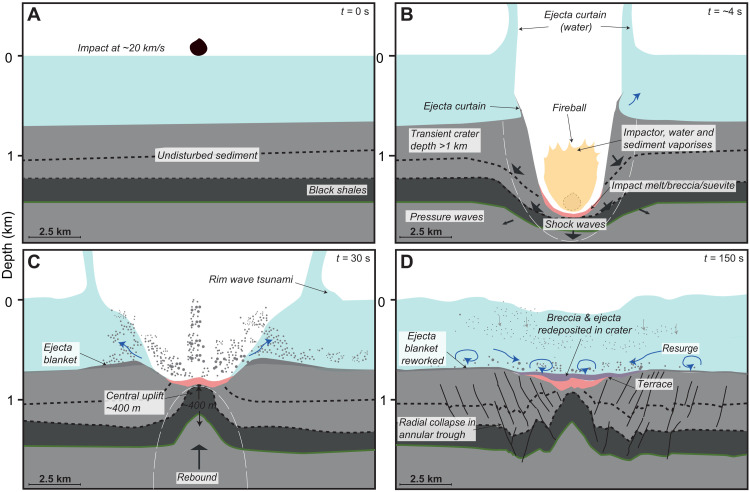
Conceptual model of the impact sequence at the Nadir impact site, based on seismic observations and analog models (*6*, *7*, *21*). (**A**) At time (*t*) = 0, the impactor hits the water surface at a velocity of ~20 km/s, initiating a rim-wave tsunami in its wake. (**B**) Several seconds later, the transient crater forms, as the impactor and a substantial body of water are vaporized. Impactites (melt rock and breccias) line the transient cavity. Tsunami waves propagate away from the evacuated crater. Shock waves cause substantial damage below and around the impact site, and seismic waves propagate across the plateau; (**C**) major uplift (~400 m relative to preimpact regional) occurs in the “rebound” crater modification phase, resulting in the formation of a raised crater peak; (**D**) radial collapse of the subsurface damage zone results in further modification of the crater, including the formation of terraces at the surface. Resurge of water transports substantial volume of ejecta and other sediment into the crater, deposited above the impactites.

Integrating seismic observations and numerical model outcomes allows us to build a conceptual model of crater formation following a hypervelocity impact (Fig. 5). The initial preimpact stratigraphy is assumed to have been near horizontal (Fig. 5A), with a >1-km-deep transient crater forming immediately after the impact (Fig. 5B). This contact/compression stage is followed by the formation of the central uplift and collapsed transient crater rims during the crater modification stage, resulting in the structural configuration observed including the final elevated crater rims and faulted terraces surrounding a central uplift. Stratigraphic features that are also consistent with an impact into a marine target include the broader area of potential ejecta deposit and/or shallow seafloor disturbance and the two-layer impactites within the crater interpreted to be unsorted and sorted breccias [e.g., (*26*–*28*)]. This conceptual model for the Nadir Crater provides a set of testable hypotheses about the formation of each of the discrete seismic units within the imaged crater, which can be tested by drilling.

### Environmental consequences of a marine impact

Numerical simulations of the impact crater combined with data from the Earth Impacts Effect Program (*46*) allow us to assess the environmental impact of the Nadir event. Such an impact would result in the instantaneous vaporization of a large quantity of water and sediment near the seabed (Figs. 4 and 5), releasing around 2 × 10^19^ Joules of energy (equivalent to ~5000 megatons of TNT). A fireball with a radius of >5 km would be produced following the impact (Fig. 5), followed by an air blast with a maximum wind velocity of ~470 km per hour at 50 km from the impact site.

Seismic waves from the impact would be equivalent to those generated by a magnitude ~7 *M*_w_ earthquake (*46*). The localized shaking of the intact seabed outside the crater would likely result in widespread fluidization of saturated sediments across a radius of several hundred kilometers, as inferred from seismic observations (Fig. 1A). Because of the greater velocity of the seismic wave, seismic shaking and liquefaction would occur first, before remobilization of seafloor sediments by tsunami waves. Seismic shaking of this magnitude would also likely result in substantial mass wasting of the margins of the Guinea Terrace, possibly triggering additional high-amplitude landslide tsunamis (*47*, *48*). An expanded set of high-amplitude reflections at the Top Maastrichtian horizon in deep water areas (Fig. 1B) may indicate that these processes occurred around the Guinea Terrace at this time.

As a result of the initial impact, a >2-km-high “ejecta curtain” (*8*) of water and sediment would form adjacent to the crater (Fig. 4). The collapse of this onto the adjacent water surface would result in the formation of a ~500-m rim wave tsunami, which would propagate away from the impact site at a rate of ~0.4 km/s. Assuming water depths are roughly equivalent in all directions, the lower portion of this rim wave resurges back into the evacuated crater, resulting in the formation of central jet of water of ~2-km height. The collapse of this jet would result in the formation of secondary collapse tsunami (*8*), propagating out from the crater again, with this process of collapse and resurge occurring several times. Both the initial (rim) and secondary (collapse) waves would be sufficient to rework sediment on the seabed across the Guinea Terrace even in water depths exceeding 800 m. This is consistent with observations of high-amplitude seismic facies interpreted as reworked ejecta and contemporaneous sediments across the Guinea Terrace (Fig. 1A). Although the rim and secondary tsunami would likely dissipate with increasing distance away from the site because of the effect of radial dampening, these waves would still likely have a major impact on coastlines in the adjacent region. This would certainly affect the West African margin and possibly also the South American margin, at some ~1000-km distance at the time of impact, where tsunami wave heights of ~5 m would be expected (*49*). On the basis of historic large eruptions, including the recent Hunga Tonga–Hunga Ha’apai volcanic eruption and the 1883 Krakatau eruption, the atmospheric pressure wave, or air blast, from such an event could also amplify the tsunami waves in the far field (*50*), resulting in substantially larger waves than predicted from models only accounting for the displacement of water by the impact.

The climatic consequences of such an event would be dependent on the quantity of volatiles/aerosols ejected into the atmosphere. In the case of Chicxulub, the impact was global due in part to the release of large quantities of sulfate aerosols from the vaporization of evaporite deposits and seawater (*30*, *51*) as well as carbonate dust (*52*) and petrogenic carbon (*53*). In the case of Nadir, we propose that substantial quantities of black carbon would be produced, particularly from the organic-rich black shale deposits of the Cenomanian and Turonian. Peak temperatures of >1300 K and a peak shock pressure exceeding ~0.5 GPa would likely convert organic matter into methane, and the instantaneous release of methane could have substantial, if short term, environmental consequences; exact quantities of volatile release would be required to assess whether this would be a regional or global effect as well as a determination of timing relative to the larger climatically active gas releases at Chicxulub. As well as thermogenic methane induced directly by the impact, a potentially much larger reservoir of methane could be liberated from gas hydrates across the Guinea Plateau and adjacent deep-water areas, due to seismic shaking and slope failure (*54*). This is a unique potential consequence of impacts in intermediate to deep water (more than several hundred meters, depending on water temperature) as it requires the presence of a thick gas hydrate stability zone (GHSZ). Accurate estimates of gas emissions require geochemical and temperature proxy data from sediments just above and below the crater floor, and accurate estimates of paleowater depth and temperature across the plateau to estimate the thickness of the K-Pg GHSZ.

Our numerical simulation combined with calculations from the Earth Impacts Effect Program (*46*) shows that the Nadir impactor would have resulted in catastrophic local-regional earthquake shaking and tsunami inundation. The Nadir impactor, which we estimate at around 400 m in diameter, is equivalent to the size of the Bennu asteroid, considered to be the most hazardous object in the near-Earth asteroid catalog, with a 1-in-1750 chance of collision in the next few hundred years (*55*). Depending on the nature of the affected terrain and proximity to population centers, an impactor of this size is predicted to result in an average of >300,000 fatalities (*56*), mostly due to the effects of the tsunami in marine target events such as Nadir.

### Age of the impact—Possible connection to Chicxulub?

The Nadir Crater is situated at or very near the K-Pg boundary, based on our seismic stratigraphic framework for the Guinea Plateau and that of previous studies (*17*). Correlation of the Top Maastrichtian (66 Ma old) seismic reflection from exploration wells in the east shows that this corresponds with the base of the Nadir Crater, although the seismic resolution results in a remaining >0.5 to 0.8 Ma age uncertainty. This suggests that the Nadir Crater, if confirmed as an impact crater, approximately coincides with the Chicxulub event in Mexico (*4*). This potential temporal coincidence with the Chicxulub event in Mexico leaves open a number of possibilities, including that (i) the Nadir impactor may have been part of a binary asteroid or have formed by partial breakup of the larger Chicxulub asteroid that led to the major K-Pg extinction event, or (ii) it may have been part of a longer-lived impact cluster, or (iii) may be causally unrelated to Chicxulub.

Dual impacts of binary asteroids have been proposed for other major Phanerozoic impact events, including the Lockne and Målingen craters in Sweden (*57*). Other proposed impact doublets (*9*) have recently been disputed, with high-precision dating of impact melts disproving a dual impact origin for the Clearwater East and West craters (*10*) and the Suvasvesi doublet in Finland (*58*). High-precision dating is absent for most terrestrial impacts (*5*), and no proposed impact doublets have been unequivocally proven to have resulted from a binary event.

Tectonic reconstructions of the North Atlantic at 66 Ma (Fig. 1) show that the Guinea Terrace was located much closer to the Chicxulub site [~5500 km; (*59*)] compared to the present day (~8000 km). Despite this, the distance between the craters is still too great a distance to have arisen from breakup of an asteroid within the Roche limit on a direct approach with Earth or atmospheric breakup before collision (*60*). However, tidal separation of a parent asteroid into two or more fragments during an earlier Earth orbit may have resulted in more widespread dispersion, with individual fragments colliding with Earth during a subsequent encounter (*61*). This is analogous to the collision of the Shoemaker-Levy 9 comet with Jupiter in 1994. The ~2-km-diameter comet initially broke apart into >20 discrete fragments as it passed within the Roche limit of Jupiter several years earlier (*62*). These collided with Jupiter over a period of about 6 days (14 Jovian days), with impact sites dispersed widely across the surface of the planet. This demonstrates that dual or multiple impacts, defined more broadly to include dispersed fragments from a parent asteroid or comet, may occur across a much wider region than that typically assumed for binary events (*9*). There is some limited evidence for other, more widely dispersed asteroids at the K-Pg boundary, including the discovery of fossil K-Pg meteorites in the North Pacific and Poland (*63*), which provides possible evidence of an asteroid shower rather than a single impactor. This would have important implications for our understanding of the K-Pg extinction event, requiring new models to be constructed for the multiple impacts and their consequences. This could include an evaluation of the interactions of regional seismic and tsunami waves from Nadir with the trans-Atlantic waves from Chicxulub over a period of hours to several days, as well as an evaluation of the aerosol and greenhouse gas contribution from each site over a period of months to years after impact (*30*).

Alternatively, if the Nadir Crater is temporally close to (±<1 Ma) but not precisely the same age as Chicxulub, it raises the question as to whether there may have been an impact cluster at the end of the Cretaceous period. Genetically related impact clusters have been proposed for the Ordovician (*12*), but other proposed clusters have not been confirmed because of a lack of sufficiently robust chronological data or because craters within proposed clusters fall within the expected terrestrial cratering rate (*2*). There are other impact craters close in age to the K-Pg boundary, particularly the large (24 km) Boltysh impact crater in present-day Ukraine, which has an age 65.4 Ma, ~650 thousand years (ka) younger than Chicxulub (*64*). Assuming a ~1500-m impactor (*64*), such an event would have an expected frequency of 1 to 2 Ma (*2*), which means that the time gap between Chicxulub and Boltysh is below the average rate of cratering for bodies of that size. There is also some limited evidence of other possible impacts, with remnants of meteorites, compositionally distinct from Chicxulub, discovered in the K-Pg boundary layer in Poland (*63*).

Estimates for the average flux of Nadir-sized impactors range from once every 100 ka (*46*) to ~700 ka (*2*). This means that, even if Nadir were confirmed to have formed within ~1 Ma, it would still not be possible to prove unequivocally the presence of a K-Pg impact cluster. However, depending on the precise age of Nadir, it could mean that there were ~3 large impact events within a short period of time near the K-Pg boundary, despite a crater preservation rate of only 15 to 25% (*5*). In this case, the impact cluster hypothesis would then require further testing by looking for evidence for other undiscovered impact craters of this age and by improving constraints on the flux of Nadir-sized bodies. As well as potential undiscovered craters, there are numerous confirmed craters of approximately Late Cretaceous to Paleogene age where absolute age constraints are absent or have large (10^6^ to 10^7^ years) uncertainty (*5*). Robust age constraints of appropriate materials from these craters would also be required to test this impact clustering hypothesis. In addition, an impact cluster might also be expected to be recorded by a flux of fine-grained extraterrestrial material in the atmosphere, as observed in the Ordovician (*13*)—this has not been previously tested. If confirmed, then the impact cluster hypothesis would have important implications for our understanding of the frequency and cascading environmental consequences of impact clusters in Earth’s history.

As with confirmation of the hypervelocity origin of the Nadir structure, the precise age of the crater and its association with the Chicxulub impact event (binary impact, impact cluster, or no causal connection) can only be tested by high-precision (±0.1% uncertainty) dating of samples obtained from Nadir by scientific ocean drilling.

## MATERIALS AND METHODS

The seismic stratigraphic framework is based on an extensive 2D seismic reflection dataset and three industry wells drilled on the southeast of the plateau (Fig. 1). The 2D data were acquired and processed by TGS and WesternGeco. The NWAAM-17 survey in Guinea and Guinea Bissau was acquired in 2017 by TGS in Guinea and Guinea Bissau; data were acquired in 2017 in water depths of 15 to 4600 meters. Acquisition parameters include a 12-km streamer length towed at a depth of 18 m, with 960 hydrophone channels of 12.5-m group length and a 25-m shot point spacing, giving a 240 full-fold data coverage. Total record length was 14 s with a 2-ms sample rate for all lines. The sound source was one 4310 in3 array towed at 8 m and firing every 25 m using a Bigshot controller system. Data were processed using a pre-stack time migration (PSTM) sequence, with a subset of data processed using a pre-stack depth migration sequence, providing data in depth and time domain. Seismic frequency at the top of the crater is around 45 Hz, and interval velocity is ~1800 m/s, giving a tuning thickness (seismic resolution) of around 10 m.

The GWG99 survey in Guinea was acquired in 1999 by WesternGeco in water depths of 150 to 4500 m. Acquisition parameters include a 6-km streamer length towed at a depth of 8 m, with 480 hydrophone channels of 12.5-m group length and a 25-m shot point spacing, giving a 120 full-fold data coverage. Total record length was 10 s with a 2-ms sample rate for all lines. The sound source was one 2000 in3 array towed at 6 m and firing every 25 m. Data were processed using a PSTM sequence, with a subset of data reprocessed in 2017 to improve imaging and reduce multiples and noise. Seismic frequency at the top of the crater is around 45 Hz, and interval velocity is ~1800 m/s, giving a tuning thickness (seismic resolution) of around 10 m.

Seismic interpretation was carried out using Schlumberger Petrel 2020 software, including horizon mapping, structural element mapping, velocity analysis, and seismic-to-well ties. Lithological, petrophysical, and biostratigraphic data from the exploration wells were used to constrain the stratigraphic age and lithology of seismic reflections. Seismic-to-well ties were based on checkshot surveys, with the tie further improved by constructing synthetic seismograms using sonic and density log data. Structural reconstructions of the preimpact stratigraphy and the geometry of the transient crater were carried out by seismic flattening of the Top Maastrichtian and KU1 horizons, respectively. On the basis of a vertical seismic resolution of ~10 m for the top of the Nadir Crater, the age uncertainty for individual seismic reflections is estimated at 0.5 Ma for the Upper Cretaceous (700 m thick deposited over 34 Ma = 20 m/Ma, 10/20 = 0.5 Ma) and 0.8 Ma for the Paleogene (400 m thick deposited over 32 Ma = 12 m/Ma, 10/12.5 = 0.8 Ma).

Minimum initial water depth estimates for the crater was estimated at 500 m from the height of the Maastrichtian clinoforms. We assume that the water depth at the top (offlap break) of the clinoforms was ~100 m. The “height” of the clinoforms from the offlap break to the base of the slope is around 300 ms TWT or ~400 m at a velocity of 2.7 km/s TWT. The actual water depth is estimated to be higher than this because of the effect of compaction and the fact that the Nadir Crater is located 50 km SW of the base of the clinoforms.

Numerical modeling of the impact was carried out using the iSALE hydrocode [(*65*) and references therein]. Hydrocodes solve the equations for conservation of mass, momentum, and energy, along with constitutive relations to describe the response of different materials to impact. The impactor was approximated as a 400-m sphere with a bulk density of 2750 kg/m^3^, impacting at 90° at an impact velocity of 20 km/s. Projectile properties are within the range expected for terrestrial meteors [(*66*) and references therein] and were chosen to create a 7- to 9-km-diameter crater based on scaling trends (*67*). Other projectile sizes, velocities, densities, etc., are possible and viable and would have been scaled to produce the same impact energy and thus the same final crater.

Parameters for the Upper Cretaceous sedimentary sequence were based on data from ODP Leg 207 on the conjugate margin (reference). We assumed that the Lower Cretaceous and Upper Cretaceous sequence is dominated by marine marl and shale and that the Cenomanian-Turonian (Top Albian to KU1) sequence is dominated by black shale deposits. Our preliminary simulations simplified the target into homogeneous weakened carbonate deposit as the constitutional model for marl is not yet available for use with hydrocodes. The material properties of the target were approximated with strength models derived from laboratory strength data and the ANEOS for calcite (*68*). This thick sedimentary target was overlain by water of varying depths—200 to 1500 m.

It is well established that the mobility of rock masses during impact crater collapse implies a dynamic strength below that expected based on static laboratory strength tests. We incorporated the Acoustic Fluidization Block Model (AFBM) to produce the necessary apparent material weakening during crater collapse (*69*) and used the AFBM parameters used for carbonates (*70*). The choice of rock strength and material weakening parameters for the models will clearly affect the final crater form, influencing both the amount of rim collapse and uplift. Of the preliminary simulations completed so far, it is likely that the material weakening of the central target is too high as the amount of uplift is larger than that shown in the seismic line across the crater. Alterations in the strength model used to better approximate the marl and shale target might produce different amounts of uplift. However, it is clear that even with different strength models, the crater morphologies created by impact into increasing sea depth follow a similar progression to that shown in Fig. 4 (*71*).
