# Supplementary material for: The Nadir Crater offshore West Africa: A candidate Cretaceous-Paleogene impact structure
Source: Sci Adv. 2022 Aug 17;8(33):eabn3096. doi: 10.1126/sciadv.abn3096 (PMC9385158; doi:10.1126/sciadv.abn3096)
Supplement: Supplementary file 1 — Figs. S1 to S4 [file sciadv.abn3096_sm.pdf]

Science Advances
AAAS

Supplementary Materials for

The Nadir Crater offshore West Africa: A candidate Cretaceous-Paleogene impact structure

Uisdean Nicholson *et al.*

Corresponding author: Uisdean Nicholson, u.nicholson@hw.ac.uk

Sci. Adv. **8**, eabn3096 (2022)
DOI: 10.1126/sciadv.abn3096

The PDF file includes:

Figs. S1 to S4
Legends for movies S1 to S5

Other Supplementary Material for this manuscript includes the following:

Movies S1 to S5

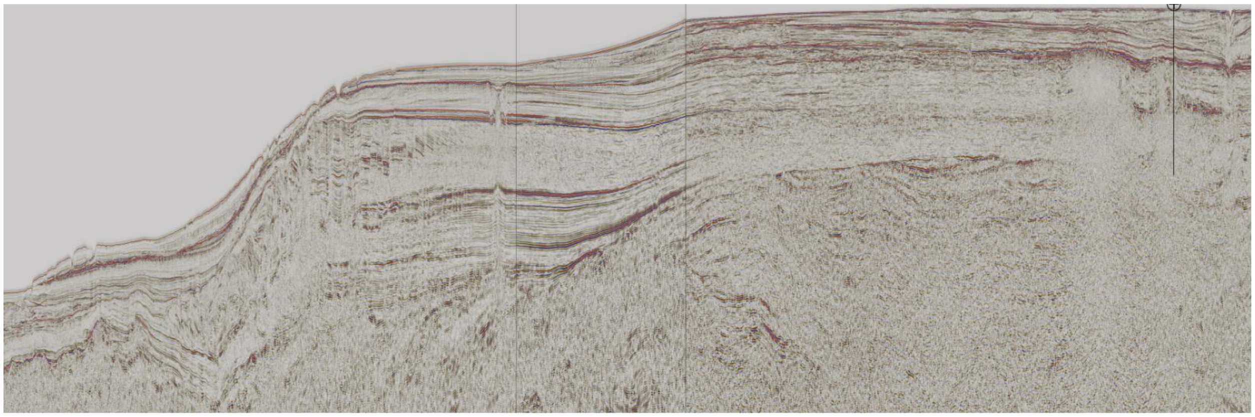

Fig S1.

Uninterpreted seismic section shown on Fig.1b

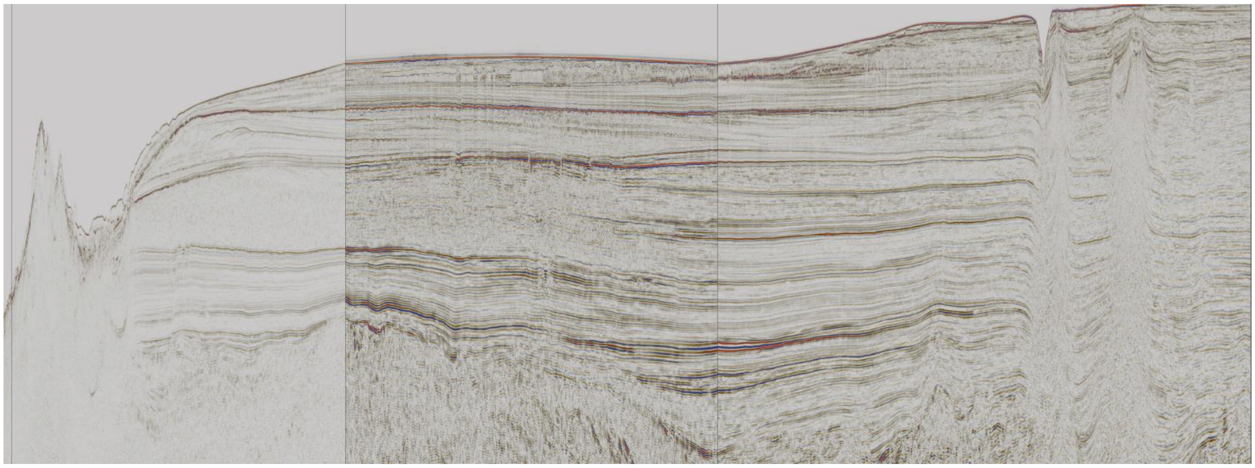

Fig S2.

Uninterpreted seismic section shown on Fig.1c

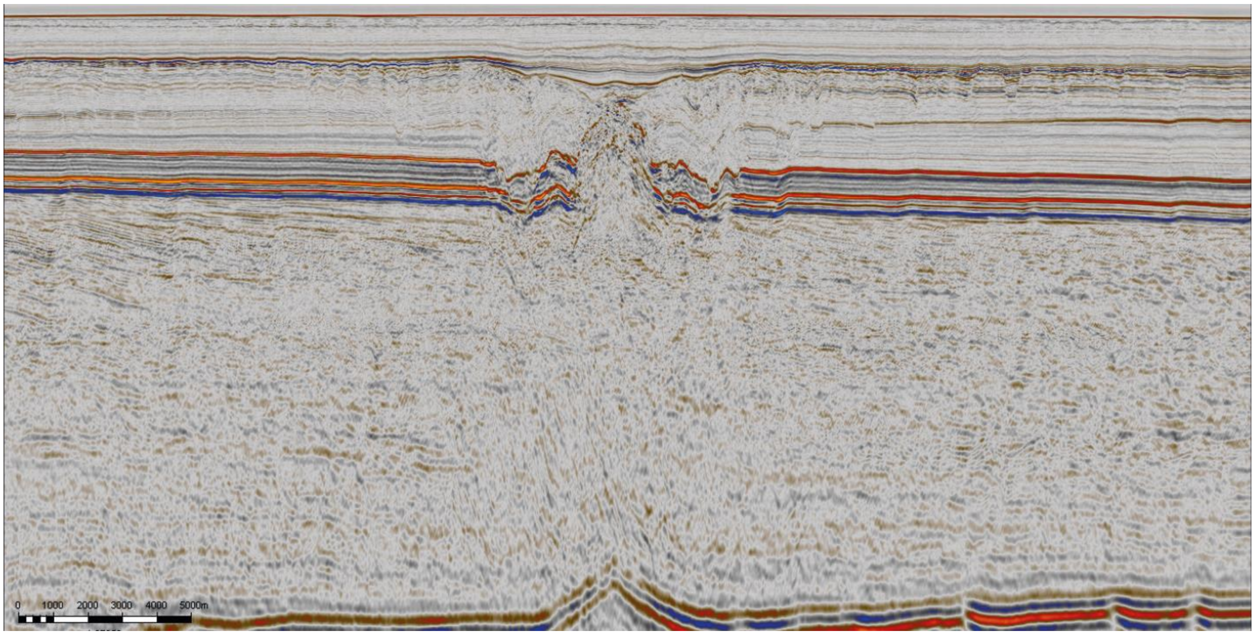

Fig S3.

Uninterpreted seismic section shown on Fig.2b

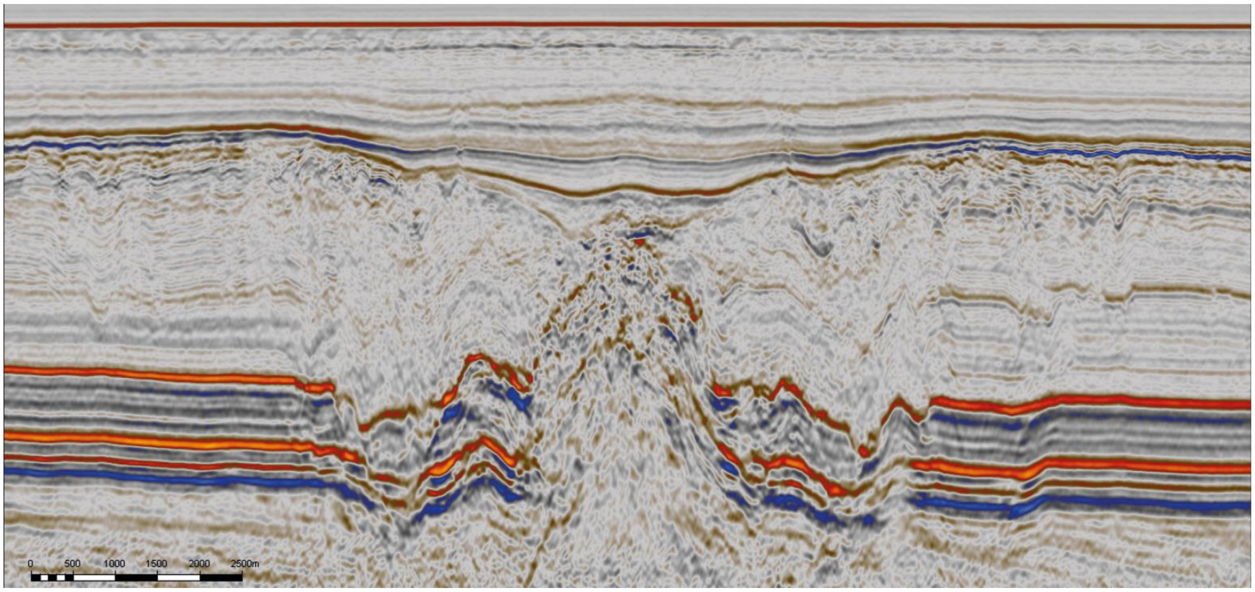

Fig S4.

Uninterpreted seismic section shown on Fig.2c

Movie S1.

iSALE hydrocode simulation of crater formation in 200 m water depth, for a 400 m diameter asteroid impacting at 90 degrees (vertical) at an impact velocity of 20 km/s.

Movie S2.

iSALE hydrocode simulation of crater formation in 500 m water depth, for a 400 m diameter asteroid impacting at 90 degrees (vertical) at an impact velocity of 20 km/s.

Movie S3.

iSALE hydrocode simulation of crater formation in 800 m water depth, for a 400 m diameter asteroid impacting at 90 degrees (vertical) at an impact velocity of 20 km/s.

Movie S4.

iSALE hydrocode simulation of crater formation in 1100 m water depth, for a 400 m diameter asteroid impacting at 90 degrees (vertical) at an impact velocity of 20 km/s.

Movie S5.

iSALE hydrocode simulation of crater formation in 1500 m water depth, for a 400 m diameter asteroid impacting at 90 degrees (vertical) at an impact velocity of 20 km/s.
